# Effect of Berberine from *Coptis chinensis* on Apoptosis of Intestinal Epithelial Cells in a Mouse Model of Ulcerative Colitis: Role of Endoplasmic Reticulum Stress

**DOI:** 10.1155/2020/3784671

**Published:** 2020-04-24

**Authors:** Shen Yan, Liu Yingchao, Wang Zhangliu, Ruan Xianli, Li Si, Ni Siyi, Zhong Jihong

**Affiliations:** ^1^Department of Gastroenterology, The Second Affiliated Hospital of Zhejiang Chinese Medical University, No. 318, Chaowang Road, Gongshu District, Hangzhou 310005, China; ^2^First Clinical Medical College of Zhejiang Chinese Medical University, No. 548, Binwen Road, Binjiang District, Hangzhou, 310051, China; ^3^Department of Pediatrics, Tongde Hospital of Zhejiang Province, No. 234, Gucui Road, Xihu District, Hangzhou 310012, China

## Abstract

The purpose of this study was to verify the effect of berberine (BBR) on endoplasmic reticulum stress (ERS) and apoptosis of intestinal epithelial cells (IECs) in mice with ulcerative colitis (UC). BALB/c mice were randomly divided into five groups as follows: blank control, model, and low-, medium-, and high-dose BBR. A dextran sodium sulfate- (DSS-) induced model of UC was prepared, and the low-, medium-, and high-dose BBR groups were simultaneously gavaged with a BBR suspension for 7 d. Disease activity index (DAI) was assessed, and tissue damage index (TDI) was assessed from colon samples after the last administration. TUNEL assays were used to detect apoptosis of IECs. Immunohistochemistry and/or real-time PCR were applied to determine the expression of GRP78, caspase-12, and caspase-3. In all BBR treatment groups, clinical symptoms of colitis and histopathological damage were significantly reduced. The high-dose BBR group exhibited particularly pronounced decrease (*p* < 0.01) in both DAI (0.48 ± 0.36) and TDI (1.62 ± 0.64) relative to the model group (1.50 ± 0.65 and 3.88 ± 0.04, respectively). In colon tissues of the model group, the number of apoptotic IECs was significantly increased; the expression of GRP78, caspase-12, and caspase-3 proteins was significantly increased; and the expression of the GRP78 mRNA was upregulated. In low-, medium-, and high-dose BBR groups, the number of apoptotic IECs was significantly reduced. Moreover, GRP78 and caspase-3 expression levels were significantly decreased in the medium- and high-dose BBR groups, caspase-12 expression was significantly decreased in the high-dose BBR group, and the *GRP78* mRNA expression level was significantly decreased in the high-dose BBR group. BBR can effectively reduce the rate of IEC apoptosis in UC mice and alleviate the inflammatory response in the colon. The underlying mechanism seems to involve ERS modulation and inhibition of ERS-mediated activation of the caspase-12/caspase-3 apoptosis signaling pathway.

## 1. Introduction

Ulcerative colitis (UC) is a chronic colonic inflammatory disease primarily characterized by mucosal inflammation and ulceration. It is often recurrent with slow recovery and is currently a refractory disorder of the digestive tract [1, 2]. In recent years, with the westernization of Chinese lifestyles and eating habits, the incidence of UC in China has continuously increased, and most patients are young or middle-aged [3]. It has been shown that damage to the intestinal mucosal barrier caused by abnormal apoptosis of intestinal epithelial cells (IECs) is the primary cause of persistent inflammatory response in UC, and activation of the caspase-12/caspase-3 apoptosis signal transduction pathway by endoplasmic reticulum stress (ERS) may play an important mediating role [4, 5]. Berberine hydrochloride (BBR) is an effective active ingredient extracted from the traditional Chinese medicine *Coptis chinensis*. It may exert a therapeutic action in UC [6], which most likely is dependent on adjustment of the intestinal flora composition [7], preservation of intestinal barrier function [8], regulation of immune response, and other protective actions [9]. Our previous studies indicated that BBR may effectively inhibit colon inflammation in UC mice by preventing the death of intestinal stem cells and destruction of tight junction proteins such as claudin-1, occludin, and zo-1, thus maintaining the homeostasis of intestinal mucosal mechanical barrier [10]. However, an exploration of the mechanisms underlying BBR treatment of UC from the perspective of regulating ERS and affecting apoptosis of IECs has not been attempted. Therefore, this study investigated whether BBR had an impact on IEC apoptosis and could attenuate UC colonic inflammatory response. Moreover, the involvement of ERS and of the subsequent activation of the caspase-12/caspase-3 signal transduction pathway in BBR action was explored.

## 2. Materials and Methods

### 2.1. Materials

#### 2.1.1. Experimental Animals

Sixty healthy 8-week-old SPF male BALB/c mice weighing 20 ± 2 g were purchased from the Shanghai SLAC Laboratory Animal Co., Ltd. (Shanghai, China), under permit number SCXK (Shanghai) 2017-0005, approval number 0345203, and laboratory animal room use permit number SYXK (Zhejiang) 2015-0008. The animal care environment was maintained at a temperature of 20–25°C and relative humidity of 40–70%.

#### 2.1.2. Drugs and Reagents

The chemicals utilized in this study were as follows: dextran sulfate sodium (DSS; MP Biomedicals, LLC, Solon, OH, USA; lot number: BJ14745, molecular weight: 5000); berberine hydrochloride (BBR; Shanghai Yuanye Bio-technology Co., Ltd., Shanghai, China; lot number: Y18D8C50814, molecular weight: 371.81); glucose-regulated protein 78 (GRP78), caspase-12, and caspase-3 primary antibodies (Abcam, Cambridge, UK; lot numbers: GR309483-1, ab62484, and ab13847, respectively); anti-rabbit and anti-mouse secondary antibody (Beijing ZSGB-BIO, Beijing, China; lot numbers: K167722B and K175212C, respectively); proteinase K (Beijing Tiangen, Beijing, China; lot number: M2011); DAB chromogenic reagent kit (Beijing ZSGB-BIO, lot number: K167722A); TUNEL *in situ* apoptosis detection kit (Roche, Basel, Switzerland; lot number: 11906800); high-purity total RNA rapid extraction kit (Shanghai Generay, Shanghai, China; lot number: 1703G01); HiScript-II Q RT SuperMix for qPCR reverse transcription kit (Nanjing Vazyme, Nanjing, China; lot number: 7E092G6); and ChamQ SYBR Color qPCR Master Mix (Nanjing Vazyme Co.; lot number: 7E092H6).

#### 2.1.3. Instrumentation

The following instruments were used in this study: a RM2235 rotary microtome (Leica, Wetzlar, Germany); TEC-2500 histopathology dryer (Changzhou Hao Silin Instrument Equipment Co., Ltd., Nanjing, China); BX43 microscope (Olympus, Tokyo, Japan); PYX-DHS500BS-II water jacket constant-temperature incubator (Shanghai Yuejin Medical Instrument Co., Ltd.); BCD-211KD3 refrigerator (TCL, Huizhou, China); C21-SDHC15K induction heater (Zhejiang Shaoxing Supor Life Electric Co., Ltd., Hangzhou, China); and 101-3 electric heating constant-temperature dryer (Shanghai Jinping Instrument Co., Ltd., Shanghai, China).

### 2.2. Methods

#### 2.2.1. Preparation of Drug Suspensions

Low-, medium-, and high-dose BBR suspensions (100, 150, 200 mg·kg^−1^, respectively) were prepared with distilled water based on human-mouse surface area conversion.

#### 2.2.2. Groups and Treatments

After one week of acclimatization, the random number table method was used to divide the animals into 5 groups of 12 mice, namely, a blank control group, a model group, and low-, medium-, and high-dose BBR groups. The UC model was established by DSS induction as previously described [11]: mice were given 5% DSS solution (5 g DSS in 100 mL of distilled water) for 7 days; the blank control group received distilled water (100 mL) *ad libitum* every day. The BBR suspension was simultaneously administered once a day by gavage to all BBR groups (administration volume: 10 mL·kg^−1^). Model and blank control groups were given an equal volume of distilled water.

#### 2.2.3. General Observations

Starting on the day of model establishment, the mental state and activity, food intake, and water intake of each group of mice were observed and recorded daily. Body weight was measured at regular intervals daily, fecal traits were examined in parallel with fecal occult blood tests, and the disease activity index (DAI) was scored as previously described [12]. DAI = (weight loss rate score + stool trait score + occult blood score)/3.

#### 2.2.4. Colon Histopathology

After drug administration, all mice were sacrificed by cervical dislocation, and colon tissue was harvested under strict aseptic conditions. The tissue was trimmed to 1.0 cm × 1.0 cm × 0.2 cm, fixed in 4% paraformaldehyde for 4 d, embedded in paraffin, and cut into serial sections of 4 *μ*m in thickness. After HE staining, histopathological changes were examined under a light microscope and the tissue damage index (TDI) was scored as previously described [13]: normal colon mucosa, 0 points; 1/3 loss of intestinal crypt glands, 1 point; 2/3 loss of intestinal crypt glands, 2 points; loss of all intestinal crypt glands, intact mucosal epithelium accompanied by mild infiltration of inflammatory cells, 3 points; and erosion and destruction of mucosal epithelium with obvious infiltration of inflammatory cells, 4 points. Ten fields of view at 200x magnification were randomly selected for each sample, and scores were recorded and tabulated.

#### 2.2.5. Detection of IEC Apoptosis in Colon Tissue

Colon tissue was cut into pieces, fixed in 4% paraformaldehyde, embedded, and sectioned. The TUNEL kit was used as per manufacturer's instructions. Briefly, the slides were deparaffinized and hydrated, while the tissue was digested with proteinase K working solution for 20 min at 37°C. Then, after rinsing with PBS, TUNEL reaction mixture (containing 5 *μ*L of TdT and 45 *μ*L of fluorescein-labeled dUTP labeling solution) and blocking solution were added, and the reaction was carried out in a humidified chamber for 30 min at 37°C in the dark. The slides were rinsed, DAB substrate was added, and color development was examined under a microscope. Hematoxylin counterstaining was performed, and the slides were dehydrated through an alcohol gradient, cleared with xylene, and sealed with neutral gum. TUNEL-positive cells appeared in brown or dark brown, and normal colonic epithelial cells appeared in blue. Five fields of view at 400x magnification were randomly selected for observation, and the number of TUNEL-positive (apoptotic) IECs was recorded and tabulated.

#### 2.2.6. Determination of GRP78, Caspase-12, and Caspase-3 Protein Expression Levels in Colon Tissue

Colon tissue was cut into pieces, fixed in 4% paraformaldehyde, embedded, and sectioned. Slides were processed according to the immunohistochemical staining protocol. Briefly, slides were deparaffinized in xylene, hydrated through an alcohol gradient, incubated in 3% hydrogen peroxide solution for 15 min at 37°C, and washed in PBS. Incubation with the primary antibody was performed overnight at 4°C. The secondary antibody was added to the slides and incubated for 30 min at 37°C. DAB staining was performed, and the progress of the reaction was observed under the microscope. Slides were rinsed, counterstained, dried, and mounted. Three fields of view at 200x magnification were randomly selected for observation, and the intensity and rate of positive expression were determined. Scoring was performed as described by Fromowitz [14]; the specific scoring criteria were as follows: (1) staining intensity (score of staining intensity exhibited by most cells minus background color): no coloring, 0 points; light yellow, 1 point; brownish-yellow, 2 points; and brown, 3 points; (2) extent of positive staining: <5%, 0 points; 5–25%, 1 point; 26–50%, 2 points; 51–75%, 3 points; and >75%, 4 points. The sum of these two measures was calculated, and a score <2 was classified as negative (−), a score of 2-3 was classified as weakly positive (+), a score of 4-5 was classified as moderately positive (++), and a score of 6-7 was classified as strongly positive (+++). This score represented the level of protein expression.

#### 2.2.7. Determination of *GRP78* mRNA Expression Level in Colon Tissue

Fluorescence real-time PCR was used for determination of *GRP78* mRNA expression. Total RNA was extracted from colon tissue using the TRIzol-centrifugal column method, the purity and content of the total RNA were determined, and cDNA was synthesized by reverse transcription as per instructions of the kit manufacturer. The PCR mixtures (20 *μ*L) consisted of 10.0 *μ*L of ChamQ SYBR Color qPCR Master Mix, 0.6 *μ*L of forward and reverse primers (10 *μ*mol/L), and 8.8 *μ*L of cDNA. PCR conditions were as follows: 95°C for 30 s, followed by 40 cycles at 95°C for 10 s and 60°C for 30 s. PCR primer sequences and amplification conditions are shown in Table 1. PCR products were electrophoresed on a 1% agarose gel and imaged using a gel imaging system. The expression of target mRNA was normalized using the mouse *β*-actin gene as an internal reference, and the relative expression level of each gene was expressed as 2 (Ct_internal reference gene_ − Ct_target gene_).

#### 2.2.8. Statistical Analysis

SPSS 20.0 statistical software was used. Continuous variables are expressed as mean ± standard deviation ($\bar{x}$ ± *s*). One-way analysis of variance was used for comparison among groups. The LSD *t*-test was used for pairwise comparisons between groups. Differences with *p* < 0.05 were considered statistically significant.

## 3. Results

### 3.1. Changes in General Conditions and Disease Activity of Mice

The disease model was successfully established, and the mice in the model group showed different degrees of mental deficit, impaired movement, and reduced food intake, accompanied by loose and bloody stool and weight loss, whereas the mental state, activity, food and water intake, stool traits, and weight of the mice in the blank control group were normal. After the end of treatment, the DAI of the high-dose BBR group was significantly lower compared to that of the model group (*p* < 0.01). The DAI scores of the low- and medium-dose BBR groups were also decreased, but the change was not statistically significant (both *p* > 0.05). The results are shown in Table 2.

### 3.2. Pathological Changes in Mouse Colon Tissue

**Figure 1:**
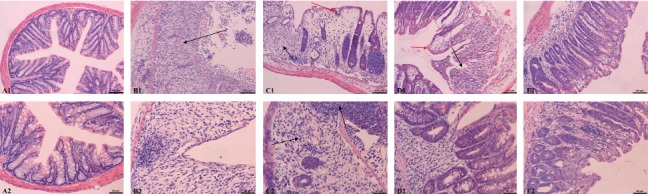
Pathological changes in mouse colon tissue (HE staining). A1–E1 (100×): blank control group; model group; low-dose BBR group; medium-dose BBR group; high-dose BBR group. A2–E2 (200×): blank control group; model group; low-dose BBR group; medium-dose BBR group; high-dose BBR group. Note. The black arrows indicate loss of glandular structure and inflammatory cell infiltration; the red arrows indicate edema of mucosal epithelium.

Microscopy showed normal colon tissue structure in the blank control group, and no lesions were found in the mucosal epithelium. In the model group, all submucosal crypt glands were lost, and inflammatory cells had infiltrated the mucosa, resulting in destroyed epithelial structure. Among the BBR groups, the colon tissue structure of the high-dose BBR mice exhibited the highest similarity to that of the normal control group. In the low-dose BBR group, there was major loss of crypt glands, submucosal inflammatory cell infiltration, and partial edema of the mucosal epithelium. The medium-dose BBR group had a reduced extent of lesions compared to the low-dose group. The TDI of the high-dose BBR group was significantly lower (*p* < 0.01) compared to the model group, whereas in the low- and medium-dose BBR groups the decrease was not statistically significant (*p* > 0.05). The results are shown in Table 2 and Figure 1.

### 3.3. Apoptosis of IECs in Mouse Colon Tissue

**Figure 2:**
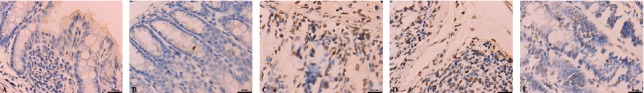
Apoptosis in mouse colon tissue (TUNEL staining, 200×). A, blank control group; B, model group; C, low-dose BBR group; D, medium-dose BBR group; E, high-dose BBR group.

To investigate apoptosis of IECs, mouse colon tissue was analyzed using TUNEL assays. TUNEL-positive cells appeared in brown or dark brown, and normal colon epithelial cells appeared in blue. In the blank control group, apoptotic cells were occasionally found in colon tissue. Moreover, the number of apoptotic colon epithelial cells in the model group was significantly higher compared to the blank control group, and the difference was statistically significant (*p* < 0.01), indicating that excessive apoptosis of IECs exacerbated the damage to the intestinal mucosal barrier. Notably, in all BBR-treated groups, the level of apoptosis was significantly lower (*p* < 0.05) compared to the model group, indicating that BBR could effectively protect IECs from excessive apoptosis in UC mice. The results are shown in Table 3 and Figure 2.

### 3.4. Expression of Markers of ER Stress-Mediated Apoptosis in Mouse Colon Tissue

**Figure 3:**
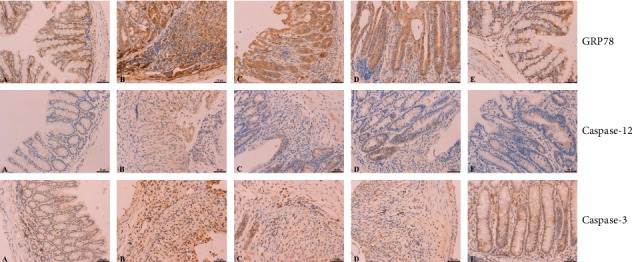
GRP78, caspase-12, and caspase-3 protein expression in mouse colon tissue (immunohistochemical staining, 400×). A, blank control group; B, model group; C, low-dose BBR group; D, medium-dose BBR group; E, high-dose BBR group.

To investigate the expression of markers of ER stress-mediated apoptosis, mouse colon tissue was analyzed using immunohistochemistry. GRP78-, caspase-12-, and caspase-3-positive cells appeared as brown or yellowish-brown, and these three proteins were mainly located in the cytoplasm. The expression of the three proteins was the lowest in the blank control group and the highest in the model group. The difference between the model group and the blank control group was statistically significant (*p* < 0.01). In high-dose BBR-treated mice, GRP78, caspase-12, and caspase-3 were significantly downregulated compared to levels in model mice (*p* < 0.01), whereas in the medium-dose BBR group, the decline was only significant for GRP78 and caspase-3 (*p* < 0.01). The results are shown in Table 4 and Figure 3.

### 3.5. Transcription of the ER Stress Marker *GRP78* in Mouse Colon Tissue

To investigate the transcription of the ER stress marker *GRP78*, mouse colon tissue was analyzed using real-time PCR. The model mice expressed a significantly higher level of *GRP78* mRNA in the colon tissue compared to that in the blank control mice (*p* < 0.05). Notably, medium- and high-dose BBR mice displayed significantly lower levels of *GRP78* mRNA in the colon tissue compared to levels in the model group (both *p* < 0.05). The results are shown in Table 5.

## 4. Discussion

ERS is a ubiquitous and highly conserved stress response mechanism in eukaryotic cells and is accompanied by a series of alterations including impaired protein synthesis and processing in the ER upon exposure to various stress conditions. Accumulation of a large number of unfolded or misfolded proteins in the ER lumen results in the imbalance of a series of intracellular processes involved in homeostasis [15]. ERS plays an important role in determining the cell fate under stress conditions, including resistance, adaptation, injury, or apoptosis. GRP78, which is located on the endoplasmic reticulum membrane, is generally considered as a molecular marker of ERS, and its expression level reflects the severity of cell stress. Under physiological conditions, GRP78 binds to ER transmembrane receptor proteins, namely, protein kinase R-like ER kinase, activating transcription factor 6, and inositol-requiring enzyme l, keeping it in an inactive state [12]. When ERS in the cell reaches a certain level, the transmembrane receptor proteins are dissociated from GRP78 and then are activated, thereby promoting protein synthesis and processing through unfolded protein response (UPR), restoring and maintaining cell homeostasis, and ultimately avoiding cell damage. However, if ERS is too strong or persistent, UPR may not be sufficient to prevent cell damage and the activated transmembrane receptor will further activate apoptosis in the stressed cells in order to maintain homeostasis [16, 17]. Caspase-12 is an initiating factor in the ERS-specific apoptosis signal transduction pathway and is only activated and phosphorylated during ERS. Under these conditions, caspase-12 may trigger the downstream factor caspase-3, thereby independently mediating apoptosis [18, 19].

IECs are a type of cell which is constantly under ERS. Under physiological conditions, moderate rates of IEC apoptosis maintain a dynamic balance between differentiation and supplementation of new intestinal cells [20]. However, excessive ERS may occur when cells are stimulated by pathogenic microorganisms in the intestinal lumen, mediators of mucosal inflammation, ischemia, hypoxia, and other stressors, thereby accelerating apoptosis and resulting in intestinal mucosal barrier damage and increased intestinal epithelial permeability [21]. Various endogenous and exogenous antigenic substances are abnormally exposed to and activate the local immune system in the intestinal tract, which causes tissue damage through cascade amplification of immune and inflammatory responses. Various studies have shown that excessive ERS can be detected in the IECs of UC patients and animal models [22–24]. Abnormal apoptosis is also common in *in vitro* models of ERS in IECs stimulated by different stressors, as well as in the colon tissue of UC patients and animal models [25–30]. Therefore, excessive IEC apoptosis following ERS is an important pathological mechanism that may result in intestinal mucosal barrier damage and UC [4, 5]. Suppressing apoptosis in IECs by inhibiting activation of the stress-specific caspase-12/caspase-3 apoptosis signaling pathway is a potential treatment strategy for UC.

BBR is an isoquinoline alkaloid extracted from *Coptis chinensis* and has strong anti-inflammatory and immunomodulatory effects. Previous studies have shown that BBR has therapeutic effects on UC [6]. However, there are few direct studies regarding the effects of BBR on ERS and IEC apoptosis. The results of the present study showed that BBR treatment could significantly alleviate clinical symptoms such as diarrhea, bloody stool, and weight loss in UC mice, as well as mitigate pathological damage in colon tissue, such as inflammatory cell infiltration in the colon mucosa, epithelial edema, destruction of epithelial structure, and loss of crypt glands. The DAI and TDI of UC mice were significantly decreased by BBR, indicating potential clinical effects of this molecule in the treatment of UC. UC mice displayed significantly more apoptotic IECs compared to normal mice. After treatment with various doses of BBR, the level of apoptosis was significantly reduced compared to the model group, indicating that excessive apoptosis of IECs is an important pathological factor in UC. The expression levels of GRP78, caspase-12, and caspase-3 were significantly increased in the colon tissue of UC mice compared to normal mice, and the relative expression of GRP78 mRNA was also increased. After treatment with medium- and high-dose BBR, the expression levels of these three proteins and of GRP78 mRNA were significantly lower than in the model group, suggesting that the ERS-mediated caspase-12/caspase-3 signaling pathway is involved in IEC apoptosis in UC mice.

In summary, BBR can attenuate UC-associated inflammatory response in the colon, most likely by controlling the ERS level and the subsequent activation of the caspase-12/caspase-3 apoptosis signaling pathway. This study contributes to the elucidation of the mechanism of action of BBR in the context of UC. However, in future, changes in the microstructure, functional proteins, and gene expression patterns of IECs under an ERS state should be studied in vitro, focusing on the key molecules involved in each step in the signal transduction process from excessive ERS triggering to abnormal cell apoptosis initiation. This will allow for the identification of novel therapeutic targets and active development of more efficient and selective ERS-blocking drugs. Despite these limitations, the future application of BBR in UC therapy is promising.

## Figures and Tables

**Table 1:** PCR primer sequences and amplification conditions.

Gene	Gene sequence number	Forward primer (5′-3′)	Reverse primer (5′-3′)	Amplicon length (bp)
GRP78	14828	ACTTGGGGACCACCTATTCCT	TCAGGAGTGAAGGCCACATAC	112
Mus*β*-actin	11461	AGAGGGAAATCGTGCGTGAC	AGGAAGAGGATGCGGCAGT	90

**Table 2:** DAI and TDI scores in mice after 7 d of treatment ($\bar{x}$ ± *s*, *n* = 12).

Group	Dose (mg·kg^−1^)	DAI	TDI
Blank control	—	0.06 ± 0.04	0.00 ± 0.00
Model	—	1.50 ± 0.65^1^	3.88 ± 0.04
Low-dose BBR	100	1.12 ± 0.53	3.58 ± 0.11
Medium-dose BBR	150	1.00 ± 0.47	2.98 ± 0.53
High-dose BBR	200	0.48 ± 0.36^2^	1.62 ± 0.64^2^

^1^ *p* < 0.01 compared to the blank control group; ^2^*p* < 0.01 compared to model group.

**Table 3:** Level of apoptosis in mouse colon tissue ($\bar{x}$ ± *s*, *n* = 12).

Group	Dose (mg·kg^−1^)	Number of TUNEL-stained apoptotic cells
Blank control	—	0.67 ± 0.12
Model	—	19.27 ± 2.01^1^
Low-dose BBR	100	9.67 ± 4.27^2^
Medium-dose BBR	150	4.00 ± 1.64^2^
High-dose BBR	200	2.07 ± 0.92^3^

^1^ *p* < 0.01 compared to blank control group; ^2^*p* < 0.05 compared to model group; ^3^*p* < 0.01 compared to model group.

**Table 4:** GRP78, caspase-12, and caspase-3 expression levels in mouse colon tissue.

Group	Dose (mg·kg^−1^)	GRP78	Caspase-12	Caspase-3
Blank control	—	2.89 ± 0.33	1.78 ± 0.67	2.22 ± 0.44
Model	—	5.78 ± 0.67^1^	4.56 ± 0.53^1^	5.33 ± 1.00^1^
Low-dose BBR	100	4.89 ± 1.17	3.00 ± 1.00	4.33 ± 0.70
Medium-dose BBR	150	4.11 ± 0.60^2^	3.44 ± 0.73	3.56 ± 1.24^2^
High-dose BBR	200	3.00 ± 0.50^2^	2.33 ± 0.50^2^	2.77 ± 1.09^2^

^1^ *p* < 0.01 compared to the blank control group; ^2^*p* < 0.01 compared to the model group. *N* = 12.

**Table 5:** *GRP78* mRNA expression levels in mouse colon tissue (relative to expression of *β*-actin).

Group	Dose (mg·kg^−1^)	*GRP78* mRNA
Blank control	—	0.347 ± 0.227
Model	—	1.065 ± 0.333^1^
Low-dose BBR	100	0.841 ± 0.504
Medium-dose BBR	150	0.642 ± 0.253^2^
High-dose BBR	200	0.599 ± 0.195^2^

^1^ *p* < 0.05 compared to blank control group; ^2^*p* < 0.05 compared to model group.

## Data Availability

The text data used to support the findings of this study are included within the article.

## Abbreviations

BBR: *Berberine*
DAI: *Disease activity index*
ERS: *Endoplasmic reticulum stress*
IEC: *Intestinal epithelial cell*
TDI: *Tissue damage index*
UC: *Ulcerative colitis.*
